# Hemostatic effect of novel carbon dots derived from *Cirsium setosum* Carbonisata

**DOI:** 10.1039/c8ra06340k

**Published:** 2018-11-09

**Authors:** Juan Luo, Meiling Zhang, Jinjun Cheng, Shuhong Wu, Wei Xiong, Hui Kong, Yan Zhao, Huihua Qu

**Affiliations:** School of Chinese Materia Medica, Beijing University of Chinese Medicine Beijing 100029 China luojuan1010@163.com bucmxiongwei@163.com; School of Basic Medical Sciences, Beijing University of Chinese Medicine Beijing 100029 China 18811790361@163.com carlosjjcheng@163.com doris7629@163.com zhaoyandr@gmail.com; Military General Hospital of Beijing PLA China 849136766@qq.com; Center of Scientific Experiment, Beijing University of Chinese Medicine 11 Beisanhuandong Road, Chaoyang District Beijing 100029 China quhuihuadr@163.com +86 10 6428 6821 +86 10 6428 6705

## Abstract

In this study, for the first time, we discovered novel water-soluble carbon dots (CDs, named CSC-CDs) from aqueous extracts of *Cirsium setosum* Carbonisata (CSC), which were uniform in size and possessed a nearly spherical shape, and the CDs exhibited low toxicity against RAW 264.7 cells by CCK-8 assay. Most importantly, tail hemorrhaging and liver hemorrhaging experiments showed that CSC-CD-treated mice had significantly shorter bleeding times than normal saline-treated mice. Coagulation assays suggested that the CSC-CDs could stimulate the extrinsic blood coagulation system and activate the fibrinogen system. These results suggested that the CSC-CDs have a remarkable hemostatic effect. The study provides evidence to support the further investigation of the considerable potential of carbon dots.

## Abstract 2

We discovered novel water-soluble carbon dots from aqueous extracts of *Cirsium setosum* Carbonisata, which have a remarkable hemostatic effect. The study provides evidence to support the further investigation of considerable potential of carbon dots.

## Graphical abstract

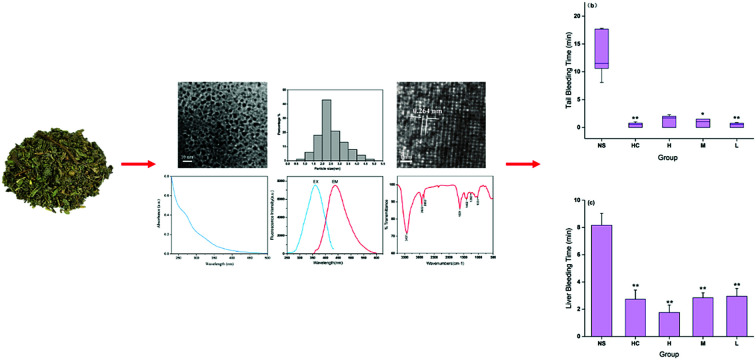

## 1. Introduction

*Cirsium setosum* (CS), belonging to the Asteraceae family (field thistle; Xiaoji in Chinese), is one of the most popular traditional medicinal herbs in China. *Cirsium setosum* Carbonisata (CSC), the charcoal processed product of CS, has been widely used as an antihemorrhagic drug in traditional Chinese medicine (TCM) for many years, which was supported by considerable clinical evidence. However, the bioactive hemostatic substances of CSC and their mechanism are not clearly reported. We tried to clarify the small molecular compounds that are responsible for the effects of CSC, but the results have been uncertain. Therefore, we focused on the novel substances generated after high temperature treatment of CS—carbon dots (CDs).

Carbon dots, as an emerging novel nanomaterial, are quasi spherical nanoparticles usually 1–10 nm in diameter.^1^ Compared to traditional semiconductor quantum dots, CDs are superior in terms of high aqueous solubility, easy functionalization, resistance to photo-bleaching, low toxicity, and excellent biocompatibility.^2–4^ In fact, the application of CDs in the biomedical domain has mainly focused on drug delivery, bioimaging, biosensors, light emitting diodes and so on.^5–7^

Recently, the activity study of carbon dots has gradually attracted scholars' attention. Das P., *et al.*^8^ synthesized zinc and nitrogen co-doped multifunctional carbon dots (N, Zn-CDs) and proved that they have an antibacterial effect. Chunduri L. A. A., *et al.*^9^ used coconut husk to formulate CDs and investigated their antioxidant efficacy. Moreover, Wang, *et al.*^10^ proved that the polysaccharide-derived CDs have strong activity to inhibit α-glycosidase and capability to reduce blood glucose level of diabetic model rats. However, the intrinsic bioactivity and potential pharmacological effects of CDs have not attracted adequate attention, and have become a domain worthy to be penetratingly explored.

Here, we discovered and purified the CDs from the water extract of CSC, and their physic-chemical properties, including particle sizes, structural details, fluorescent behaviors, elemental composition, and surface functional groups were thoroughly characterized. Furthermore, we assessed the cytotoxicity of CSC-CDs and their antihemorrhagic effects, as well as the related hemostatic mechanisms.

## 2. Experimental

### 2.1 Animals

Male Kunming mice (weighing 30.0 ± 1.0 g) and Sprague-Dawley rats (weighing 190.0 ± 10.0 g) were purchased from the Laboratory Animal Center, Si Beifu, with a Laboratory Animal Certificate of Conformity. Animals were housed in an environmentally controlled breeding room (temperature, 24.0 ± 1.0 °C; relative humidity, 55–65%; and a 12.0 h light/dark cycle). The animals were fed a standard diet (diet for small laboratory animals M1) and given water *ad libitum*. The animal experimental design and protocols used in this study were approved by the Ethics Review Committee for Animal Experimentation at the Beijing University of Chinese Medicine. All the experimental procedures were performed in accordance with the Regulations for the Administration of Affairs Concerning Experimental Animals approved by the State Council of People's Republic of China.

### 2.2 Chemicals

The CS was purchased Beijing Qiancao Herbal Pieces Co., Ltd., and the CSC was prepared in our laboratory, as described in Section 2.3. Hemocoagulase (HC) for injection was purchased from Jinzhou Ahon Pharmaceutical Co., Ltd., (Liaoning, China). Dialysis membranes with a molecular weight cut-off (MWCO) of 1000 Da were purchased from Beijing Ruida Henghui Technology Development Co., Ltd., (Beijing, China). The cell counting kit CCK-8 was purchased from Dojindo Molecular Technologies, Inc., (Kumamoto, Japan). Pentobarbital sodium and other analytical grade chemical reagents were obtained from Sinopharm Chemical Reagents Beijing (Beijing, China). All water used was distilled.

### 2.3 Preparation of CSC-CDs

**Fig. 1:**
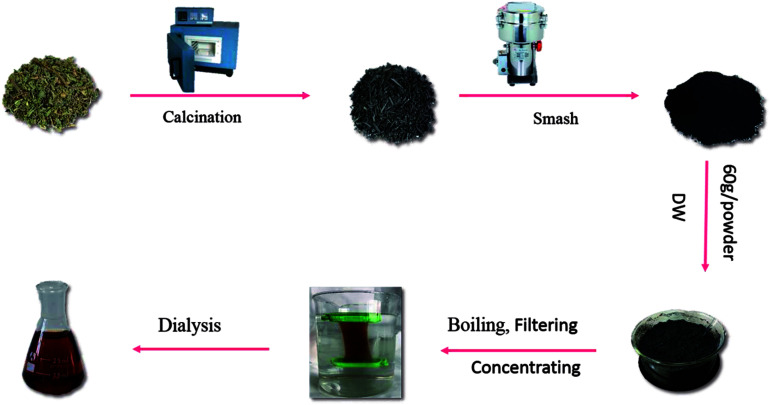
Flow diagram of the preparation of *Cirsium setosum* Carbonisatus-carbon dots (CSC-CDs).

The CS was placed in a crucible, which was covered with aluminum foil before replacing the lid to form a seal and was calcined using a muffle furnace (TL0612, Beijing Zhong KeAobo Technology Co., Ltd., China) at 350 °C, for 1 hour. Finally, the carbonized herbs (namely CSC) were pulverized into a fine powder after cooling to room temperature. Then, the CSC was dissolved in deionized water and boiled two times for 2.0 h each and the mixture was filtered to remove the precipitates and concentrated. The concentrated CSC solution was purified by dialysis (molecular weight cutting off of 1.0 kDa) to remove other impurities, which was dialyzed for 5 days. The Fig. 1 showed entire preparation process for the CSC-CDs.

### 2.4 High-performance liquid chromatography (HPLC) study of CSC-CDs and CS

Comparison the components of the purified CSC-CDs aqueous solution and a methanol extract of CS were performed on an Agilent 1260 HPLC instrument (Agilent, Waldbronn, Germany), comprised of a quaternary pump, diode-array detector, autosampler, and column compartment. A Reliash-C_18_ column (250 mm × 4.6 mm, Orochem, Illinois, USA) packed with 5 mm octadecyl-bonded silica (C_18_) was used for separation of the CDs. The detection condition was modified according to a previously published procedure.^11^ Methanol and water were used as the mobile phase in gradient mode as follows: 0–10 min, 10% to 40% methanol; 10–20 min, 40% to 60% methanol; after 20 min, 60% methanol. The flow rate was 1.0 mL min^−1^ and the column temperature was 25 °C. The detection wavelength was set at 254 nm and the injected sample size was 10 μL.

### 2.5 Characterization of CSC-CDs

TEM was performed using a Tecnai G220 electron microscope (FEI Company, USA) operating at 200 keV. The structural details and atomic lattice fringes of CSC-CDs were examined by HRTEM (JEN-1230, Japan Electron Optics Laboratory Japan). Fourier-transform infrared spectroscopy (FTIR) (Thermo, California, USA) was performed using KBr pellets in the range of 4000–400 cm^−1^. Absorption and fluorescence measurements were acquired using a UV-visible double beam spectrophotometer (CECIL, Cambridge, UK) and a fluorescence spectrophotometer (F-4500, Tokyo, Japan). The surface composition and chemical environments of the CDs were recorded using X-ray photoelectron spectroscopy (XPS) (ESCALAB 250Xi, Thermo Fisher Scientific, USA) using excitation with the mono X-ray source, Al Kα (1486.6 eV).

### 2.6 Quantum yield measurements

Quantum yield determination was achieved according to a previously established procedure by comparing integrated fluorescence intensities. Quinine sulfate in 0.1 M H_2_SO_4_ (quantum yield 54%) was chosen as the standard, as previously described.^12^ The absorbance of the aqueous solution of carbon dots and reference sample were kept below 0.10 at 360 nm.

The quantum yield was calculated using the following equation:*Q*_c_ = *Q*_q_(*A*_c_/*A*_q_)(*I*_q_/*I*_c_)(*η*_c_^2^/*η*_q_^2^)where the subscript ‘‘c” and ‘‘q’’ refer to CSC-CDs and quinine sulfate, respectively, *Q* is the fluorescence quantum yield, *A* is the absorbance at the excitation wavelength, and *I* is the refractive index of the solvent.

### 2.7 Cytotoxicity test of CSC-CDs

RAW 264.7 cells were cultured in Dulbecco's modified Eagle's medium (DMEM) containing 20% fetal bovine serum (FBS), at 37 °C, 5% CO_2_, and 95% humidity. Briefly, cells were seeded in 96-well plates at a density of 1 × 10^5^/mL, with 100 μL medium per well. After incubation for 24 h, the original medium in each well was discarded and 100 μL of medium containing various concentrations of CSC-CDs was added to the designated wells. The controls were treated with medium only. Then, 10 μL of CCK-8 was added to each well. The plates were incubated for 3 h at 37 °C and the absorbance at 450 nm was detected with a microplate reader (Biotek, Vermont, USA).

### 2.8 Hemostasis studies of CSC-CDs

To determine whether CSC-CDs are the hemostatic substance in CSC, the mouse tail amputation and liver scratch models were used according to previously published methods.^13–15^ In brief, Kunming mice were divided into the following groups and subcutaneously injected as indicated: negative control (normal saline, NS); positive control (HC, 0.67 kU kg^−1^); and high-, medium-, and low-dose CSC-CDs (8.33, 3.33, and 1.67 mg kg^−1^, respectively). The mouse were anesthetized with 4% pentobarbital sodium *via* intraperitoneal injection (50.0 mg kg^−1^). In the tail amputation model, the mouse tails were transected using a sterile scalpel at the point where the tail diameter was 1.10–1.14 mm (approximately 10 mm from the tip). Then, each tail was immediately placed on filter paper. In the liver scratch model, liver injury was established by scratching the left lateral lobe with a 2 mL syringe, causing the liver to bleed, and the incision was then overlaid with filter paper. In both models, the bleeding was monitored at 30 s intervals until hemostasis was achieved.

### 2.9 Measurement of coagulation parameters

A total of 40 Sprague Dawley rats (weighing 190.0 ± 10.0 g) were randomly divided into the following five groups (*n* = 8/group) and subcutaneously injected as indicated: negative control (NS); positive control (HC, 0.67 kU kg^−1^); and high-, medium-, and low-dose CSC-CDs (8.33, 3.33, and 1.67 mg kg^−1^, respectively). Two hours after the subcutaneous injections, blood samples were withdrawn from the aorta abdominalis and then placed in plastic tubes with 3.2% sodium citrate (citrate : blood: 1 : 9, v/v) for 15 min. Then, the samples were centrifuged at 3000 rpm for 15 min to obtain plasma. Routinely, coagulation parameters, including the values of activated partial thromboplastin time (APTT), prothrombin time (PT), thrombin time (TT), and fibrinogen (FIB), were measured using an automatic coagulation analyzer.^16^

### 2.10 Statistical analysis

Software package for statistical analysis (SPSS) version 17 is employed for data entry and analysis. The normally distributed and variance homogeneity data are presenting as the (mean ± standard) deviation (SD). Multiple comparisons were performed using a one-way analysis of variance (ANOVA) followed by the least significant difference (LSD) test. The non-normally distributed data are expressed as medians (quartile ranges). The values of *P* < 0.05 denotes significant and *P* < 0.01 denotes highly significant results.

## 3. Results

### 3.1 HPLC analysis study

**Fig. 2:**
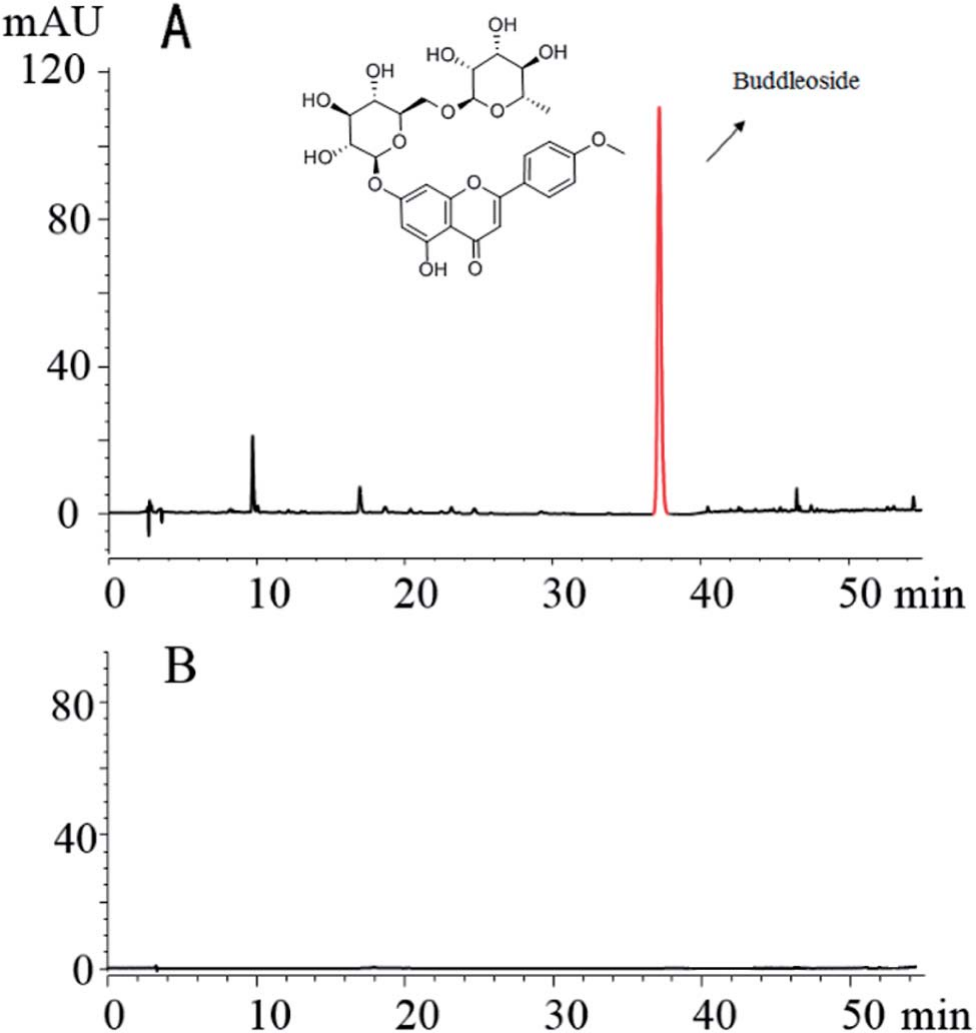
High-performance liquid chromatography fingerprint of CS (A) and CSC (B).

The fingerprint analysis shows that the methanol extract of CS contained several compounds, such as buddleoside. In contrast, the small molecule compounds in the CS were not found in the CSC dialysis solution. The results of this study show that small molecules are virtually absent after charcoal processing and dialysis of the CS (Fig. 2).

### 3.2 Characterization of the CSC-CDs

**Fig. 3:**
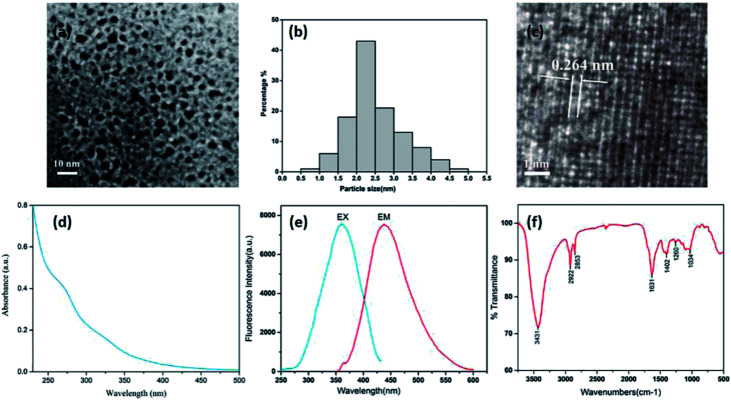
Characterization of CSC-CDs. (a) Transmission electron microscopy (TEM) images of (CSC-CDs) displaying ultra-small particles. (b) TEM size distribution of CSC-CDs. (c) High-resolution TEM (HRTEM) image of CSC-CDs. (d) Ultraviolet-visible spectrum. (e) Fluorescence spectra for both excitation (EX) and emission (EM). (f) Fourier-transform infrared spectrum.

Fig. 3(a) shows a TEM image where the CDs can be clearly seen. The CDs are uniform in size, possess a nearly spherical shape, and have a uniform dispersion without apparent aggregation. They have a narrow size distribution between 1.0 and 5.0 nm, with a maximum population at 1.0–3.0 nm and a mean diameter of (2.6 ± 0.7) nm (Fig. 3(b)), as determined by statistical analysis of more than 100 particles by using ImageJ software. Furthermore, the HRTEM shows that the CDs had a lattice spacing of 0.264 nm (Fig. 3(c)). The luminescent properties of the CSC-CDs were investigated next. The CSC-CDs have a weak absorption band at ∼269 nm, which was attributed to the π–π* electron transition (Fig. 3(d)). In addition, the fluorescence emission spectrum of the CSC-CDs show a strong peak at 438 nm, when excited at 360 nm (Fig. 3(e)). The quantum yield was 4.55% with an excitation wavelength of 360 nm, using quinine sulfate as the reference.

The surface chemical characteristics of the CSC-CDs were determined by FTIR spectroscopy. As shown in Fig. 3(f), the presence of O–H group was indicated by the strong characteristic absorption at 3431 cm^−1^ and the characteristic absorptions at 2922 cm^−1^ and 2853 cm^−1^ indicate –CH_3_ (stretching) and –CH_2_ (stretching), respectively. The intense band at approximately 1631 cm^−1^ was attributed to the C=O group, and the peak at 1402 cm^−1^ indicates the stretching vibration of C–N and C=C bonds. In addition, the peaks at approximately 1034 cm^−1^ are indicative of the C–O stretching vibrations of CSC-CDs.^17,18^

**Fig. 4:**
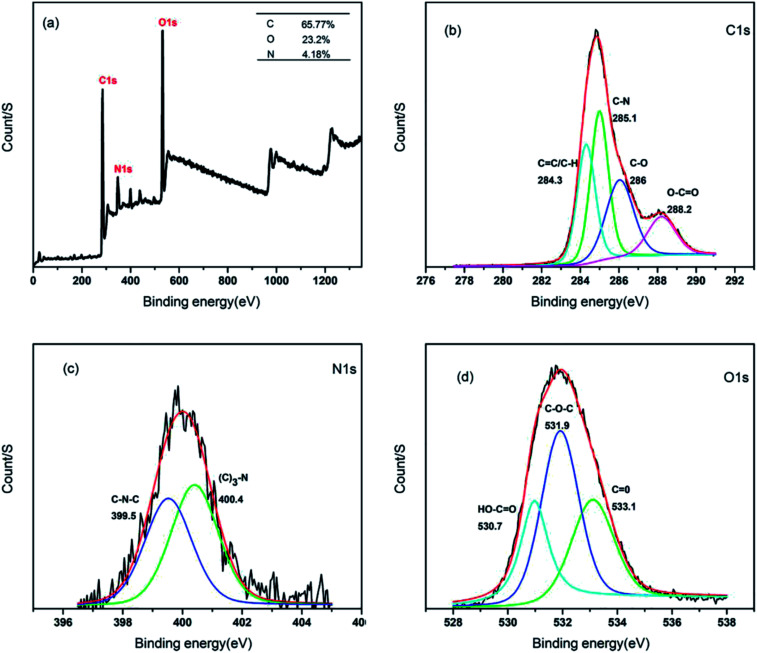
Characterization of CSC-CDs. (a) XPS survey spectrum of CSC-CDs; (b) high resolution XPS spectrum of C 1s region; (c) high resolution XPS spectrum of N 1s region; (d) high resolution XPS spectrum of O 1s region.

In order to determine the surface composition and chemical environments of the CSC-CDs, XPS was used. In the XPS survey spectrum of the CSC-CDs, as shown in Fig. 4(a), the peaks at 284 eV, 400 eV, and 531 eV correspond to the elements of C 1s, N 1s, and O 1s, respectively. This spectral result indicates that the CSC-CDs are composed of carbon, nitrogen, and oxygen with atomic percentages of 65.77%, 4.18%, and 23.20%, respectively. The four different peaks in the C 1s spectrum correspond to the sp^2^/sp^3^ carbon for C=C at 284.3 eV, C–N at 285.1 eV, C–O at 286.0 eV, and O–C=O at 288.2 eV, as shown in Fig. 4(b). The high-resolution spectrum of N 1s corresponds to the chemical environments for C–N–C at 99.5 eV and (C)_3_–N at 400.4 eV (Fig. 4(c)). Furthermore, the high-resolution scans of the O 1s region indicated the different chemical environments of HO–C=O at 530.7 eV, C–O–C at 531.9 eV, and C=O at 533.1 eV (Fig. 4(d)). Thus, the XPS spectra revealed that the CSC-CDs are carbon-rich with both oxygen and nitrogen atoms.^19–21^

### 3.3 Cytotoxicity test of CSC-CDs

**Fig. 5:**
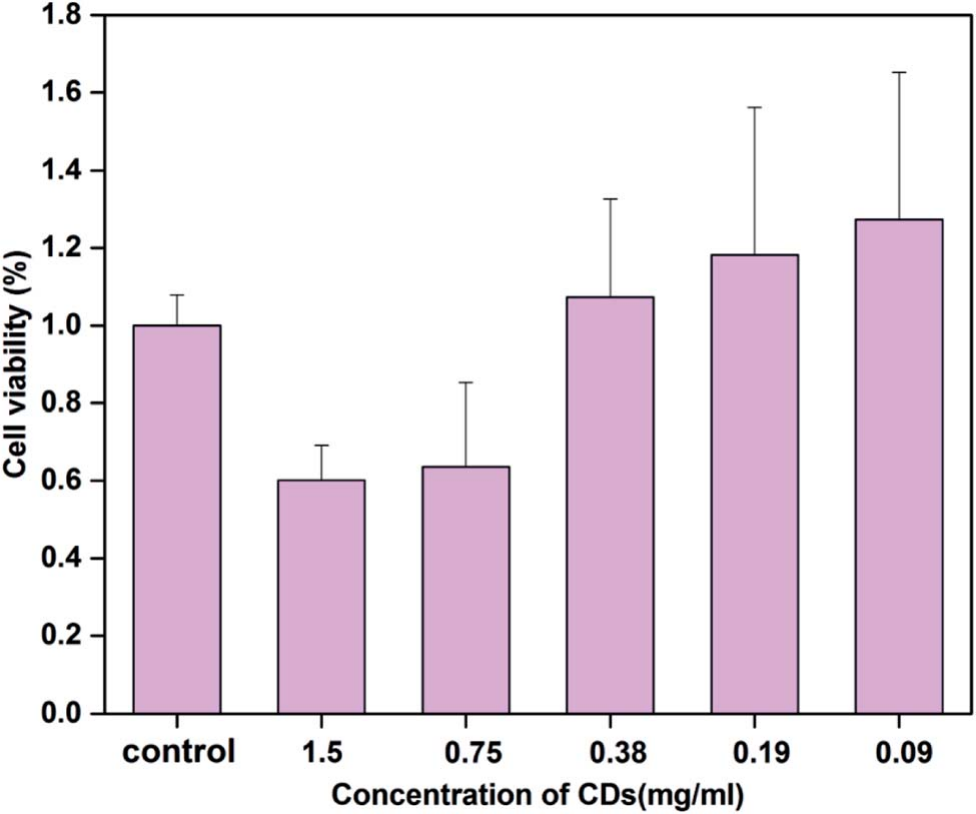
Cell viability of RAW 264.7 cells after incubation with various concentrations of CSC-CDs for 24 h. Cell viability was assessed using the CCK-8 assay.

The toxicity of CDs has always been an important issue in their biological application. Cell cytotoxicity experiments of CSC-CDs were evaluated using RAW 264.7 cells lines through the CCK-8 assay. As shown in Fig. 5, the obtained carbon dots show no apparent toxicity to the cells, even though the carbon dots concentration was increased to 0.38 mg mL^−1^.

### 3.4 Hemostasis study

**Fig. 6:**
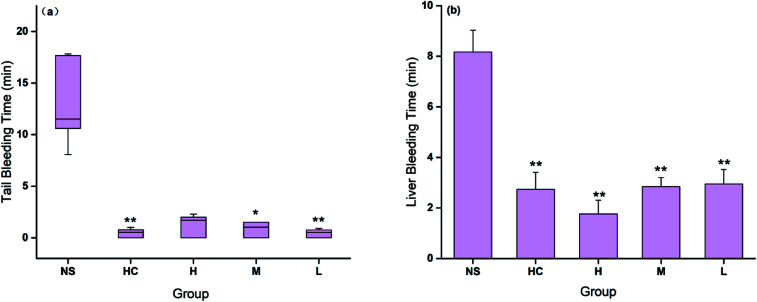
(a) Time of hemostasis in Kunming mouse tail amputation model; (b) time of hemostasis in Kunming mouse liver scratch model. There were five groups in each model: normal control group (NS), hemocoagulase group (HC), and different concentrations of CSC-CDs groups. *P < 0.05, **P < 0.01.

The hemostatic effect of CSC-CDs were evaluated by both injury models. The tail bleeding time decreased in all CSC-CDs-treated groups compared with that in the control group. Fig. 6(a) showed that the bleeding time in the low-dose CSC-CDs-treated mice was similar to that in the positive control (HC)-treated mice and was remarkably lower than that in the normal mice (NS, *P* < 0.01). The bleeding times in the medium-dose and high-dose CSC-CDs-treated mice were also shorter than that in the normal mice (NS, *P* < 0.05).

Furthermore, in the liver scratch model, the high-, medium-, and low-dose CSC-CDs, and the HC treatment significantly decreased (*P* < 0.01) the liver bleeding time (1.76 ± 0.54, 2.84 ± 0.36, 2.94 ± 0.57, and 2.73 ± 0.67 min, respectively) compared with that of the control group (NS, 8.17 ± 0.86 min). No significant difference was observed between the CSC-CDs and HC groups (Fig. 6(b)).

### 3.5 Hemostatic mechanism study

**Fig. 7:**
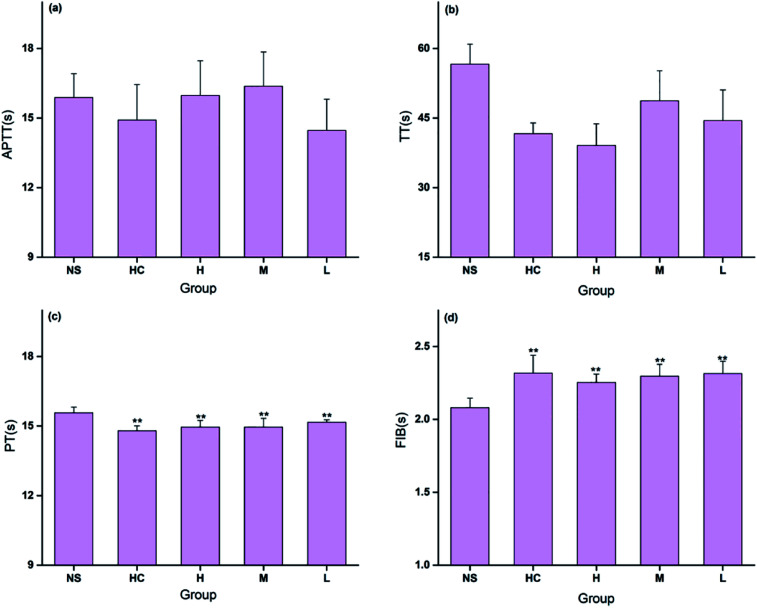
Effects on coagulation parameters. (a) Activated Partial Thromboplastin Time (APTT); (b) Thrombin Time (TT); (c) Prothrombin Time (PT); (d) Fibrinogen (FIB). There were five groups (*n* = 8/group): normal control group (NS), Hemocoagulase Group (HC) and different concentrations of CSC-CDs groups. *P < 0.05, **P < 0.01.

The coagulation parameters PT, APTT, TT, and FIB were evaluated using mouse plasma to determine the hemostatic mechanism of the CSC-CDs. As shown in Fig. 7, the TT and APTT values were not significantly different among the five treatment groups. All doses of CDs and the positive control drug decreased PT significantly (*P* < 0.01). Meanwhile, all doses of CDs and the positive control drug increased FIB significantly (*P* < 0.01). The blood clotting process includes the intrinsic and extrinsic coagulation pathways. In this study, the PT decreased and the FIB content increased in the CSC-CDs groups, whereas the APTT and TT values showed no significant difference compared with the corresponding values of the control group. These observations indicated that the CSC-CDs had positive effects on the extrinsic coagulation pathway and may be relevant to activating the fibrinogen system.

## 4. Discussion

CSC and other charcoal drugs have a long history of clinical applications for the treatment of hemorrhagic disorders in Traditional Chinese Medicine (TCM). However, the hemostatically active substances are still obscure. These reports that used CDs obtained from various herbs and plant materials^22–24^ are the motivation for our exploration of CSC-CDs prepared from CSC.

Contrasting the composition changes of CS and the aqueous extract of CSC-CDs by HPLC analysis, and it is found that the aqueous extract of CSC-CDs were virtually absent small molecules, which ruled out the interference of small molecules in the hemostatic efficacy animal experiment. In the TEM images, it can be clearly seen that the particle size and morphology of CSC-CDs is consistent with previously reported of CDs.^25–27^ The atomic lattice fringes of the CSC-CDs are similar to that of graphene quantum dots.^28^ The results of XPS and FTIR show that the structure of CSC-CDs contains hydroxyl groups and carbonyl groups, and the photoluminescence of CD can be attributed to the multiphoton activity process from various oxygen-containing functional groups.^25^ This phenomenon was consistent with previous results reported for CDs.^29^ In addition, the UV-vis data were consistent with those in a previous report of graphene quantum dots formed by the carbonization of cellulose-based materials.^28,30^ The unique emission properties of CDs may originate from the size of the CSC-CDs, availability of sp^2^ sites, aromatic conjugation, and defects in the structure.^25^ On the basis of the above characterization, we concluded that CSC-CDs were obtained from CSC by heating at 350 °C.

To determine whether CSC-CDs has hemostatic effect, mouse tail amputation and liver scratch models were used to assess the hemostatic effects of CSC-CDs. These two models are useful and commonly used *in vivo* assays, which do not require specialized equipment. Our results demonstrated that HC (positive control) and CSC-CDs treatments were highly effective in controlling the hemorrhage compared to that of NS treatment (control group) in both bleeding models. For the first time, the hemostatic effect of CSC-CDs was validated by both tail amputation and liver scratch mouse models.

The coagulation process was complicated and possesses extrinsic, intrinsic, and common coagulation pathways. Therefore, four indicators, specifically PT, TT, APTT, and FIB, were selected to study the effects of CSC-CDs on the coagulation system. This can identify alterations of the extrinsic (mainly reflected by PT), intrinsic (mainly reflected by APTT), and common (reflected by all four indicators) coagulation pathways. In this study, the PT decreased and the FIB content increased in the CSC-CDs groups, whereas the APTT and TT values showed no significant difference compared to the values in the control group. These results indicated that the CSC-CDs beneficially affected the extrinsic coagulation pathway and that may be relevant to the activation of fibrinogen. This study was a preliminary evaluation of the hemostatic activity of the CSC-CDs and further investigations are needed to elucidate the underlying mechanisms of these effects. The above results suggested that CSC-CDs possesses a remarkable hemostasis property, which provides evidence to support the further investigation of the considerable potential hemostatic drug and effective material basis of TCM.

## 5. Conclusions

This study showed the presence and physic-chemical properties of CDs in CSC. The results of the CCK-8 assay indicated that the inherently low toxicity of CSC-CDs allow them to be used in biological applications. The CSC-CDs exhibited moderate hemostatic activities in the mouse tail amputation and liver scratch models and these effects may be associated with extrinsic coagulation activity and activation of the fibrinogen system, according to the evaluation of the mouse coagulation parameters. The study not only provided a novel strategy to explore the material basis of CSC, but also opened the door for the CDs as good candidates for more practical biomedical applications.

## Conflicts of interest

The authors declare that they have no conflicts interests.

## Abbreviations

CS: *Cirsium setosum*
CSC: *Cirsium setosum* Carbonisatus
TEM: Transmission electron microscopy
HRTEM: High-resolution transmission electron microscopy
TCM: Traditional Chinese medicine
CDs: Carbon dots
MWCO: Molecular weight cut-off
CCK-8: Cell counting kit 8
DW: Deionized water
HPLC: High-performance liquid chromatography;
FTIR: Fourier-transform infrared spectroscopy
XPS: X-ray photoelectron spectroscopy
PL: Photoluminescence
DMEM: Dulbecco's modified Eagle's medium
FBS: Fetal bovine serum
APTT: Activated partial thromboplastin time
TT: Thrombin time
PT: Prothrombin time
FIB: Fibrinogen
HC: Hemocoagulase

## Supplementary Material
